# A One Health Zoonotic Vector Borne Infectious Disease Family Outbreak Investigation

**DOI:** 10.3390/pathogens14020110

**Published:** 2025-01-23

**Authors:** Edward B. Breitschwerdt, Ricardo G. Maggi, Charlotte O. Moore, Cynthia Robveille, Rosalie Greenberg, Emily Kingston

**Affiliations:** 1 Intracellular Pathogens Research Laboratory, Comparative Medicine Institute, College of Veterinary Medicine, North Carolina State University, Raleigh, NC 27607, USA; rgmaggi@ncsu.edu (R.G.M.); comanvel@ncsu.edu (C.O.M.); cmrobvei@ncsu.edu (C.R.); emkingst@ncsu.edu (E.K.); 2 Medical Arts Psychotherapy Associates, P.A., 33 Overlook Road, Suite 406, Summit, NJ 07907, USA; rgmd@verizon.net

**Keywords:** *Bartonella*, *Babesia*, flea, tick, vector, neuropsychiatric, infection, digital PCR

## Abstract

This study reinforces the value of a One Health approach to infectious disease outbreak investigations. After the onset of neuropsychiatric symptoms in their son, our investigation focused on a family composed of a mother, father, two daughters, the son, two dogs, and a rabbit, all with exposures to vectors (fleas and ticks), rescued dogs, and other animals. Between 2020 and 2022, all family members experienced illnesses that included neurological symptoms. Prolonged menorrhagia (130d) in the youngest daughter ultimately resolved following antibiotic administration. One dog was diagnosed with a splenic hematoma and months later spinal histiocytic sarcoma. The father, both daughters, and one dog were seroreactive to multiple *Bartonella* spp. antigens, whereas the mother and son were not seroreactive. *Bartonella quintana* DNA was amplified from specimens obtained from all family members. Based upon DNA sequencing, infection with *B. quintana* was confirmed for the mother and both pet dogs. *Bartonella henselae* DNA was amplified and sequenced from the youngest daughter, the son, and one dog (co-infected with *B. quintana*), and from *Ctenocephalides felis* collected from their pet rabbit. All five family members and one dog were infected with *Babesia divergens*-like MO-1. Both parents were co-infected with *Babesia microti.* Droplet digital PCR supported potential infection with a *Borrelia* species in three family members. This study provided additional case-based evidence supporting the role of stealth *Babesia*, *Bartonella*, and *Borrelia* pathogens as a cause or cofactor in neurological and neuropsychiatric symptoms. We conclude that a One Health investigation approach, particularly for stealth vector borne pathogens such as *Babesia*, *Bartonella*, and *Borrelia* spp., will enhance clinical and epidemiological understanding of these organisms for animal and human health. During outbreak investigations it is critical to document travel and vector exposure histories, symptoms, and pathology in pets and human patients, contact with rescued, wild, or feral animals and perform diagnostic testing that includes family members, pets, and vectors.

## 1. Introduction

One Health emphasizes a collaborative approach to problem-solving that involves consideration of animal health, human health, and environmental factors that impact the health of all living organisms [1,2,3]. In the context of infectious disease epidemiology, a One Health approach was of critical importance throughout the SARS CoV2 pandemic [3,4,5]. During and after the pandemic, the many interfaces between animals and humans became rigorous areas of epidemiological, clinical, and laboratory studies, while at the same time environmental spillover of the virus from humans to animals, such as cats, deer and rats were unanticipated outcomes of this “human” pandemic [5,6]. Acute epidemic or pandemic illnesses clearly lend themselves to a One Health problem solving approach. As illustrated by this outbreak investigation, utilization of a One Health approach for the study of more chronic and insidious infections can also generate information that would not be achieved by focusing on illness in a single mammalian species. Unfortunately, infrastructures that support a One Health approach for the study of chronic illness outbreaks remain limited to nonexistent.

Isolation, nucleic acid amplification, and antigen detection modalities provide direct diagnostic evidence to support infection with one or more pathogens [7,8,9]. However, such approaches have often proven to be of insufficient sensitivity in the case of chronic infection with stealth pathogens, a group of organisms that are highly skilled at evading the immune system, surviving treatment despite antibiotics, and producing prolonged infection [10,11]. *Babesia*, *Bartonella*, and *Borrelia* spp. are stealth pathogens that are primarily vector-borne and can infect a diverse array of mammalian species [8,9,10,11]. Although members of these three genera have most often co-evolved with a specific vector that transmits the organism to a specific reservoir host, incidental or opportunistic infection can occur across mammalian species [12]. With the increased sensitivity of new diagnostic testing modalities, incidental infections and co-infections with stealth pathogens are being detected more frequently than previously appreciated. This study documents infection with multiple stealth pathogens in family members and their pets, thereby reinforcing the value of a One Health approach to infectious disease outbreak investigations. It is important to acknowledge that the microbiological findings documented during this single point in time investigation could not clarify when or how these infectious agents were acquired or the role they may or may not have played in the symptoms reported by family members or the clinical findings in their pets.

## 2. Outbreak Investigation: Study Participants, Testing Methods, Pets, and Flea Vectors

**Figure 1:**
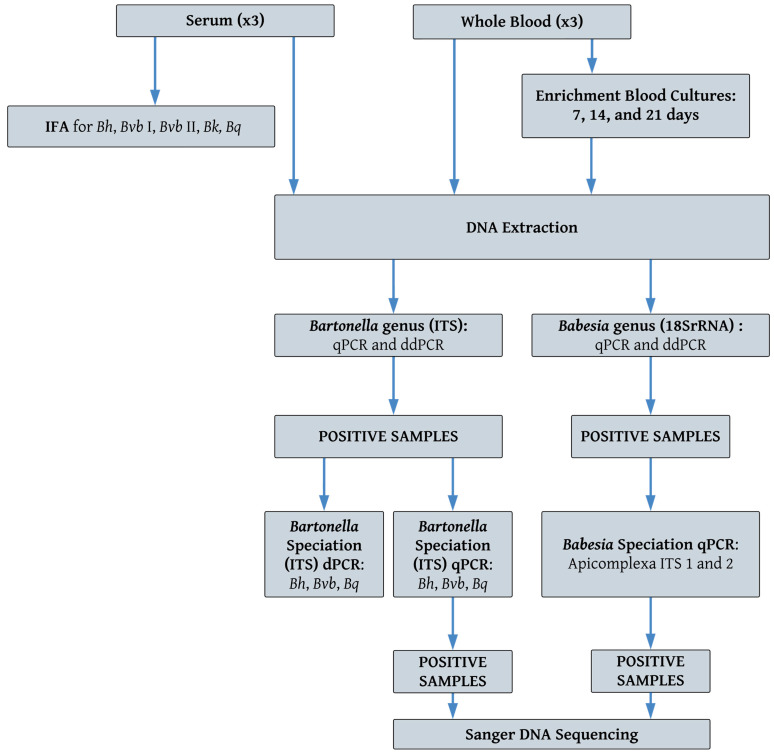
Microbiological testing approach used in this study. *Bh*: *Bartonella henselae*; *Bvb* I or II: *Bartonella vinsonii* subsp. *berkhoffii* genotype I or II; *Bk*: *Bartonella koehlerae*; *Bq*: *Bartonella quintana*.

This investigation was initiated after the mother emailed the corresponding author in December 2021 regarding her son’s illness. Her email began with the following sentences: “I am writing to you today with what may be considered a long shot. I have read some of your articles and as a desperate mother I feel compelled to reach out. I have a 10-year-old son that became very, very ill in May of 2021. Over the course of about a week he developed extremely severe OCD, complete defiance (ODD), rages that left holes in the wall, constantly dilated pupils, severe separation anxiety, burning sensations in his feet, shooting pains, random rashes, pain behind his eyes, he was shaking his hands, spinning in circles, motor and vocal tics, hallucinations, and the list goes on”. In August 2022, all five family members (father, mother, two daughters, and a son), residing in northeastern Ohio, were enrolled in a research study entitled: Detection of *Bartonella* Species in the Blood of Healthy and Sick People (NCSU Institutional Review Board approval, IRB 1960). Previously described *Bartonella* serological, enrichment blood culture, qPCR, and *Babesia*/*Bartonella*/*Borrelia* droplet digital PCR (BBBddPCR) and digital PCR (dPCR) methods were used to test all family members, their two dogs (Dog 1 and Dog 2), and pet rabbit [8,13,14,15]. The testing approach used in this study is depicted in Figure 1. All human family members underwent three blood draws on alternating days (15, 17, and 19 August 2022). The serum sample from the first blood collection day was tested by *Bartonella* spp. indirect fluorescent antibody (IFA) assays. DNA from whole blood and serum from all three days was extracted and tested by qPCR, dPCR, and ddPCR, as well as whole blood being inoculated into liquid enrichment culture for DNA sampling at 7, 14, and 21 days. DNA extracted from whole blood, tissues obtained at surgery and autopsy from one pet dog (Dog 1), and fleas removed from the family pets (dogs and rabbit) were also tested by *Babesia/Bartonella/Borrelia* qPCR, ddPCR, and dPCR.

## 3. Human Study Participants

**Figure 2:**
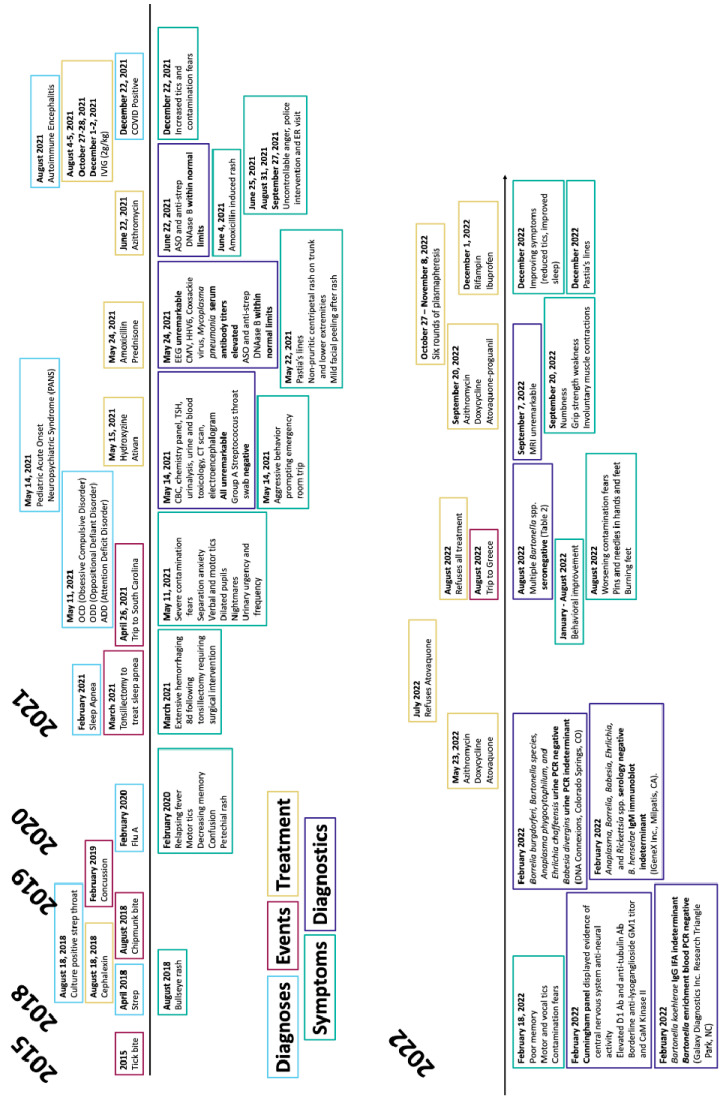
Historical timeline for the youngest son who was 10 years old at the time of this investigation in August 2022. His illness and neurological symptoms reportedly predated the onset of neurological symptoms in other family members.

The son, born in August 2011, had a facial dermoid cyst removed at 5 months of age. At 20 months of age, a pulpectomy was required to repair a severe enamel defect (hole through tooth). Following a flu-like illness in February 2020, fever of unknown origin persisted for 2 months. Although several abnormal behavioral events were documented earlier in their son, definitive neurological manifestations began in May 2021, with psychiatric diagnoses including obsessive–compulsive disorder (OCD), oppositional defiant disorder (ODD), and attention deficit disorder (ADD). Figure 2 details the son’s historical symptoms, diagnoses, animal and vector exposures, diagnostic testing, and treatments. Despite substantial diagnostic testing, no etiology was identified as a cause of the child’s symptoms prior to the infectious disease research testing reported below.

During the ensuing months of the son’s illness, the father and oldest daughter developed fatigue, insomnia, headaches, and neurological symptoms (Table 1). The father was previously diagnosed with major depressive disorder in 2015 and cervical spinal misalignment in August 2021. Except for ocular migraines, the mother, who had prior diagnoses of scoliosis, cutaneous basal cell carcinoma, endometriosis, and increased ocular pressures, remained relatively asymptomatic. At 24 months of age, the oldest daughter developed pneumonia and had an intense allergic reaction (generalized hives), thought to be associated with amoxicillin administration or viral infection. The father, mother and oldest daughter had not been tested diagnostically or treated empirically for suspected vector borne infection. Beginning at age 2, the youngest daughter developed multiple episodes of nursemaid’s elbow (radial head subluxation), and subsequently hyperflexible joints, recurrent headaches, neck pain, postural orthostatic tachycardia syndrome (POTS)-like symptoms, dyslexia, dysgraphia, and sensory hypersensitivity (to touch). Following her first menstrual cycle in February 2022, she developed severe menorrhagia with protracted bleeding that continued for 130 consecutive days. Detailed evaluation by her pediatrician, a pediatric hematologist, and an endocrinologist, including an assessment of clotting parameters, genetic and/or endocrine abnormalities did not determine causality. The girl was placed on birth control pills, up to three times the usual dosage, plus intermittent use of tranexamic acid on heavier bleeding days, but none of these interventions stopped the intense blood flow. Given her brother’s history of multiple vector exposures, she was tested for evidence of vector-borne infections that might have been contributing to her medical problems, especially the severe menorrhagia. She was non-seroreactive for *Anaplasma*, *Babesia*, *Ehrlichia*, and *Rickettsia* spp. Western immunoblot testing for *Borrelia burgdorferi* identified two IgM bands (23 and 41 kDa), but no IgG bands. *Bartonella elizabethae* IgM immunoblot was indeterminant, whereas *Bartonella* genus and *Bartonella vinsonii* IgG immunoblot results were reported as positive (IGeneX Inc., Milpatis, CA, USA). When tested two weeks later, the youngest daughter was *Bartonella quintana* IgG seroreactive (1:128) by IFA (non-seroreactive to the other three *Bartonella* spp.) and enrichment blood culture qPCR was negative (single blood draw, Galaxy Diagnostics Inc. Research Triangle Park, NC, USA). Based upon the *Borrelia* and *Bartonella* serology results, and the protracted menstrual bleeding, treatment with doxycycline and azithromycin was initiated. Although bleeding resolved completely within two weeks of antibiotic administration, both antibiotics were continued for an additional 6 weeks, until research blood collection in August 2022. After antibiotic discontinuation, abnormal uterine bleeding did not recur.

## 4. Family Travel History, Animal, and Vector Exposures

The family, including the dogs, had traveled extensively throughout the United States (Midwest, Southeast, Northeast, and West Coast), with documented yearly tick attachments to the dogs. All family members had experienced numerous mosquito and sandfly bites at a second home in South Carolina. Between 2018 and 2020 the family visited the Amazon rain forests in Peru, the Bahamas, Canada, and Mexico. In the context of animal exposures, all family members reported bites or scratches by pet cats, dogs, rabbits, birds (poultry), and pet rodent bites. Foxes, coyotes, owls, hawks, eagles, and domestic dogs and cats were reportedly prevalent in the neighborhood. Insect exposures for all family members included fleas, ticks, biting flies, mosquitoes, spiders, and honeybees. The father and the mother reported head louse exposure as a child (approximately 1990) and scabies mite parasitism after attending a Public Health conference in December 2005 in Philadelphia, respectively.

## 5. Puppy Fostering

After the son developed OCD, ODD, and ADD in May of 2021, the parents chose to foster puppies as an animal-assisted therapeutic measure for their son. From August to November 2021, the family fostered 12 litters of puppies ranging in age from 5 weeks to 4 months, which were rescued from Kentucky, Ohio, Alabama, and North Carolina and maintained in the household for short periods (1–5 wks) prior to adoption. Most litters were around 8 weeks of age, had received the first vaccination series, and had been treated for internal and external parasites. Following a three-day quarantine, fostered puppies were introduced into indoor and outdoor areas occupied by family pets. Rabbits were always maintained in areas that were not accessible by the dogs. As is the case with many foster situations, both the adoptees and family pets experienced flea and tick infestations, various dermatological conditions including sarcoptic mange, and intestinal parasitism with hookworms, roundworms, tapeworms as well as giardiasis and parvovirus enteritis. A severe flea infestation, most likely originating with foster puppies from a farm in Ohio, persisted from July through September 2022, despite attempted interventions in the household and the application of preventive products for the pets.

## 6. Flea Exposures

In July 2022, the two pet rabbits (6.5 and 5.5 years of age, respectively), which were housed in the owner’s basement and transiently in an outdoor enclosure, as well as both pet dogs were treated with an acaracide for a severe flea infestation. Weeks later, the older rabbit was found dead, and no autopsy was performed. The 10-year-old female and 8-year-old female basset hounds developed cutaneous allergies for the first time; both diagnosed with atopy, and treated with oclacitinib (Apoquel Tablets, Zoetis LLC, Parsippany, NJ, USA). Family members experienced flea bites, particularly when entering the basement, where the rabbits were housed. In September 2022, fleas were collected from pet dogs, rabbits, a current foster puppy, and from the family’s household for *Bartonella* and *Rickettsia* qPCR testing and flea phylogenetic clade analysis via the *cox*1 gene [16]. Fleas selected for testing from the dogs (*n* = 2), rabbits (*n* = 5), and the household (*n* = 5) were determined to be *Ctenocephalides felis* (the cat flea), while all fleas from the foster puppy (*n* = 5) were *Ctenocephalides canis* (the dog flea). *Bartonella henselae* DNA was amplified from one *C. felis* collected from a rabbit, while all other fleas were *Bartonella* spp. qPCR negative. All *C. felis* were infected with *Rickettsia asembonensis* and were assigned to previously defined Clade 4 [16]. *Rickettsia* spp. DNA was not amplified from *C. canis*.

## 7. *Babesia*, *Bartonella*, and *Borrelia* Microbiological Testing of Pet Dogs and a Rabbit

In August 2022, the younger basset hound (Dog 1) was clinically diagnosed with a large intra-abdominal mass. A computer tomography (CT) scan confirmed a large (26 by 28 cm) cavitary lesion within the spleen that was removed by splenectomy. Fleas were noted on the dog at the time of surgery. A splenic hematoma was diagnosed histologically. Blood, serum, and frozen and formalin-fixed tissues (normal skin, splenic mass, alesional spleen) were collected during surgery. The dog was *B. henselae* (1:128) and *Bartonella koehlerae* (1:64) seroreactive but was not *B. vinsonii* subsp. *berkhoffii* (1:32) seroreactive. *Bartonella henselae* DNA was qPCR amplified and sequenced from non-lesional splenic tissue, whereas the splenic hematoma tissue was *Bartonella* qPCR negative. *Bartonella quintana* DNA was amplified and sequenced from non-lesional skin and from a 14-day enrichment blood culture, indicating co-infection with two *Bartonella* species (Table 2). The dog was treated with a combination of doxycycline and enrofloxacin for 6 weeks. Subsequently, during the fall of 2022, the dog developed posterior paresis that progressed rapidly to near paralysis. An MRI identified a mass impinging on the lumbar spinal cord. Following humane euthanasia and autopsy, a histiocytic sarcoma was diagnosed in the liver and spinal mass, which was confirmed with the immunohistochemical markers CD18 and iba1. Retrospective testing of DNA extracted from this dog’s blood documented infection with *B. divergens*-like MO-1.

*Bartonella quintana* DNA was also amplified and sequenced from the older, healthier basset hound (Dog 2, Table 2). *Bartonella henselae* DNA was amplified from fleas found on the pet rabbit, and *Bartonella* DNA was amplified from the rabbit’s seven-day enrichment blood culture. The sequence obtained from this sample was not adequate to determine the species. *Babesia divergens*-like MO-1 DNA was not amplified from the healthy dog or the rabbit.

## 8. *Bartonella* Serology and *Babesia*, *Bartonella*, and *Borrelia* Molecular Testing for Family Members

Prior to the August 2022 testing, the son had been diagnosed by more than one physician as suffering from a Pediatric Autoimmune Neuropsychiatric Disorder Associated with Streptococcal Infections (PANDAS), Pediatric Acute-Onset Neuropsychiatric Syndrome (PANS), and autoimmune encephalopathy. He had been treated with a variety of medications, including amoxicillin, azithromycin, doxycycline, atovaquone, and valacyclovir but nonetheless remained quite ill. *Bartonella* spp. IFA serology results for the five family members obtained in August 2022 are summarized in Table 3. The mother and son were non-seroreactive to any of the five *Bartonella* spp. antigens tested. The father and both daughters were *B. henselae* seroreactive at titers greater than or equal to 1:128. The father and one daughter were also seroreactive to *B. quintana* at titers of 1:128.

Based upon *Bartonella* qPCR, dPCR, and ddPCR testing of DNA extracted from blood, amplification at low levels were achieved for the father, mother, both daughters, and the son. Despite being non-seroreactive to all five *Bartonella* spp. IFA assays, *B. quintana* (121/127 bp, 95.3% similarity to GenBank accession number LC031779) was successfully sequenced from the mother’s 7-day enrichment blood culture. *Bartonella henselae* DNA was successfully amplified and sequenced from the youngest daughter (106/106 bp, 100% homology with *B. henselae* SA2 GenBank# AF369529) and the youngest son (104/104 bp, 100% with *B. henselae* SA2 GB# AF369529) from enrichment blood cultures at 7 and 14 days, respectively.

To identify the infecting *Bartonella* species from samples that were genus dPCR or ddPCR positive, but qPCR negative, dPCR was performed using species specific probes as previously described [17]. In total, 21 different blood, serum, and enrichment culture extracted DNA samples were tested, with two to six samples (selected on the basis of dPCR positive droplets) tested per family member. For each family member, *B. quintana* was amplified from at least one sample, and in everyone other than the youngest daughter, *B. quintana* was amplified from more than one sample. Additionally, *B. henselae* DNA was also amplified from the oldest daughter and the son. *Bartonella vinsonii* subsp. *berkhoffii* genotype I, II, and III DNA was not amplified from any of the 21 samples.

Using multiple primer sets targeting the *Babesia* ITS1 and ITS2 regions, all five family members were infected with *B. divergens*-like MO-1 (Table 4). In addition, both parents were co-infected with *Babesia microti.* Based upon *Borrelia* ddPCR testing of blood and enrichment blood cultures, the father (1 drop), mother (2 drops), and oldest daughter (1 drop) were potentially infected with a *Borrelia* species. Efforts to confirm the species by DNA sequencing were not successful. All DNA extraction, enrichment culture, and PCR negative controls remained negative during *Babesia*, *Bartonella*, and *Borrelia* testing.

## 9. Discussion

Strikingly, based upon DNA amplification or DNA sequencing, all family members and one dog were infected with *B. divergens*-like MO-1, both parents were infected with *B. microti*, and all family members, both dogs, and one pet rabbit were infected with *Bartonella* (*B. quintana* and/or *B. henselae*, or an undetermined species). Based upon droplet digital PCR results, three family members may have been infected with a *Borrelia* species. Both dogs were infected with *B. quintana*, one of which was coinfected with *B. henselae* and *B. divergens*-like MO-1. To our knowledge, an infection with *B. divergens*-like MO-1 has not been reported previously in a dog. A flea from a rabbit was infected with *B. henselae.* In a recent case series, we reported infection with *Babesia odocoilei* in seven individuals, six of whom were co-infected with *Bartonella* spp.; two co-infected with *B. henselae*, three co-infected with *B. henselae* and *B. quintana*, and one co-infected with *B. henselae*, *B. quintana* and *B. vinsonii* subsp. *berkhoffii* [12]. When and how co-infections with a *Babesia* and *Bartonella* spp. occurs remains clinically and epidemiologically unclear. Acquiring co-infections with these genera from a specific vector seems less likely than acquiring individual infections at different time points from different vectors, from animal bites or scratches, or by other modes of transmission. For example, a child most likely acquired *B. henselae* from a feral cat scratch at two years old, followed by tick transmission of *B. divergens*-like MO-1 and *B. odocoilei* at 4 years of age, resulting in the microbiological documentation of co-infection with three pathogenic organisms at 8 years old [18]. Recent advances in molecular-based diagnostic modalities, most importantly droplet digital or digital PCR, were primarily responsible for the direct detection of stealth vector borne pathogens that can cause long standing intravascular infections reported in this and previous studies [12,17,18,19]. Although the clinical and pathological consequences of occult, undiagnosed co-infections with *Babesia* and *Bartonella* species are yet to be determined, co-infections can contribute to complex disease presentations, a lack of targeted antimicrobial therapy, and poor patient outcomes [20,21,22].

Historically, the human body louse (*Pediculus humanus humanus*) was considered the sole vector for transmission of *B. quintana* [23]. Although vector competence remains to be determined, *B. quintana* DNA has been documented in head lice, cat fleas, rodent fleas, and pigeon mites [24,25]. Based upon a systematic review and meta-analysis, arthropods from 28 of 37 countries contained *B. quintana* DNA, further indicating that *P. humanus humanus* is unlikely to be the sole competent insect vector [23]. Bloodborne *B. quintana* was documented in a woman who was bitten by a feral cat and months later *B. quintana* DNA was amplified from the feral cat’s enrichment blood culture [26]. Infection with *B. quintana* was found in 15% (30 of 200) of cat blood samples obtained in northwest Iran [27]. In addition to human patients, *B. quintana* endocarditis has been reported in dogs [28]. Infection with *B. quintana* has been relegated to poor hygiene, homeless individuals, and those afflicted with war, famine, and natural disasters [23,24,25]. Although this family had traveled extensively, there was no recent history of pediculosis or body louse infestations, suggesting that another mode(s) of transmission was involved. From a One Health perspective, both dogs were infected with *B. quintana*, potentially serving as reservoirs for vector or dog bite transmission, as reported for putative dog bite transmission of *B. vinsonii* [29]. The fact that this family rehomed dogs from distant states on a temporary basis emphasizes the potential for vector or potential horizontal transmission to the family and their pets.

Infection with stealth pathogens is often associated with incredibly low pathogen levels that can be beyond the detection limits of qPCR, conventional PCR, and Next Generation Sequencing-based diagnostic assays [20,21,22]. Also, for some pathogens, definitive markers of infection (e.g., specific antigen or target RNA/DNA molecules) are not currently available. The combination of multiple direct detection diagnostic assays used in this investigation, including enrichment blood culture combined with detection of pathogen DNA by qPCR, dPCR, and ddPCR, followed by species specific probe dPCR, and/or DNA sequencing, provided unprecedented increases in diagnostic sensitivity. Based on amplification of pathogen gene sequences, this combined culture, digital PCR and species–specific probe approach facilitated direct detection of *Babesia* and *Bartonella* pathogen DNA in pets and family members [8,14,30]. Without using these more targeted diagnostic techniques, amplification and sequencing of *B. divergens*-like MO-1 DNA from one dog and all five human family members would not have been possible, as the 18SrRNA qPCR results were consistently negative or equivocal (i.e., unconfirmed by DNA sequencing). Signal analysis of the qPCR sequence product obtained from the mother’s 7-day enrichment blood culture contained mixed sequencing chromatograms (double peaks with one chromatogram as a low signal). These overlapping signals in some regions of the amplified DNA indicated the potential for alternative allele sequences (*Bartonella* co-infection) as described previously for *Babesia* co-infection [30]. Clinically and epidemiologically, a lack of diagnostic sensitivity results in the under-diagnosis of stealth pathogens, limiting our ability to definitively identify or define disease pathologies, adversely affecting treatment decisions. Clinicians, researchers, patients, and the National Institutes of Health Tickborne Disease Taskforce have all called for the development of diagnostic tests that directly detect antigens, RNA, or DNA of stealth pathogens [9,31]. Unlike serological tests or patient immune profiling assays, which provide indirect evidence of exposure, direct testing modalities provide clinicians with more definitive evidence of infection. While increases in diagnostic sensitivity are promising, direct detection techniques remain complex and continue to have sensitivity limitations. In the author’s opinion, primarily due to issues with sensitivity, direct testing modalities cannot displace or replace indirect testing modalities at this time, thus both modalities should be used concurrently in the diagnostic and epidemiological settings.

As visually encapsulated in the graphical abstract, the One Health investigational approach used in this study documented similar vector-borne bacterial and protozoal pathogens among human and dog family members that were temporally associated with flea infestations, exposures to tick vectors, and exposure to rescued dogs. Our findings re-emphasize three important contemporary considerations in the context of disease outbreak investigations: (1) the increased rapid movement of animals, people, and vectors throughout the world, (2) the need for more sensitive direct diagnostic detection assays, and (3) the importance of educating human and veterinary clinicians to have a One Health, global mindset, particularly when investigating varied or nonspecific symptoms among family members [1,2,3]. Climate change in conjunction with the increased speed of transport in our modern world has continued to remove barriers, including those that historically prevented or limited zoonotic pathogen transmission [32,33]. The results of this One Health investigation highlight several important concepts relative to environments that are closely shared by animals, humans, and vectors, all having important implications for the health of families and their pets. To comfort their sick son, the parents elected to rehome dogs from multiple states within the central and southeastern United States—locations in which there are plentiful insect and arthropod vectors and the pathogens they transmit. The benefits of fostering and transporting dogs around the country or world for adoption are clear; however, the risks associated with pathogen relocation and transmission are often ignored or limited during rescue efforts. In 2021, an international working group noted the ongoing spread of parasites and vectors by relocating dogs and cats from distant sites [34,35]. The group provided specific recommendations to medical professionals regarding the importance of vector borne and zoonotic disease surveillance, as well as the need to diagnose and treat internal and external parasites when rehoming dogs [34]. The results of this investigation further emphasize the One Health implications of critically assessing animal contacts from diverse geographic locations, vector exposures, and the travel histories for both pets and people. In addition, a One Health approach to vector borne disease prevention is of critical importance. To the extent possible, people and pets should avoid situations in which vector exposure is likely to occur; humans should wear clothing and use repellents that prevent tick attachment and should routinely administer safe and effective acaracide products to prevent infestations on their pets.

Human and veterinary medical training must adapt to our increasingly mobile and global society by providing instruction on disease-causing organisms that are not typically found in the area where the individual is educated. Unlike disease surveillance systems for humans, domesticated animals and wildlife have no centralized or government-supported surveillance systems, despite the increasing number and diversity of household pets and their proximity to family members. As of 2024, there are 89.7 million pet dogs and 73.8 million pet cats in the United States [36]. Over 50% of households in the United States contained one or more pet(s), most commonly dogs (60%) and cats (42%). With the movement of pets indoors over the past 50 to 100 years, the exposure of humans to various disease-causing organisms, regularly infecting or colonizing pets is of greater concern, with humans sharing their tables, counter tops, sofas, and beds with their pets [32]. Although somewhat ubiquitous throughout the world, mammalian exposure to *C. felis* and *B. henselae* are closely associated with warm, humid, climates, such as the southeastern United States [16,37]. Infection with *B. henselae* was sequenced from two of three children, one dog, and fleas removed from the rabbit. A clinician obtaining a thorough history from this family would recognize frequent international travel, as well as repeated exposures to dogs fostered from flea-endemic regions of the United States. Efforts to recognize and communicate disease outbreaks and risk, particularly within individual households and local communities, are of utmost importance.

Although controversial and potentially overlapping in pathogenesis, based upon published criteria, pediatric acute-onset neuropsychiatric syndrome (PANS) and pediatric autoimmune and neuropsychiatric disorders associated with streptococcal infections (PANDAS) were appropriate diagnoses for the son in this investigation [38]. PANS is a broader diagnostic category that does not denote an etiology in the title but can be associated with infections, metabolic disturbances, or other causes of neuroinflammation [39]. PANDAS specifically occurs following a streptococcal infection; however, if or the extent to which occult infection with stealth intravascular pathogens contributes to PANDAS has not been intensively investigated. In both disorders it is thought that the individual’s immune system mistakenly attacks healthy brain cells, specifically in the basal ganglia area, resulting in autoimmunity. Symptomatically, children with PANS or PANDAS exhibit a sudden onset of symptoms that may include personality changes, obsessive-compulsive symptoms, tics, restricted eating behavior, sensory hypersensitivity, mood changes, and cognitive changes. Repeat streptococcal exposure results in repeated flairs of neuropsychiatric symptoms. If, or the extent to which, stealth infection with *Babesia*, *Bartonella,* or *Borrelia* species might be a cofactor in PANDAS deserves additional research consideration. Infection with *Bartonella* spp. has been more recently associated with PANS, schizophrenia, psychosis, and neuropsychiatric symptoms, with a subset of patients simultaneously developing cutaneous lesions [17,40,41,42]. Despite months of antimicrobial drugs, the son remained infected with *B. divergens*-like MO-1, had both *B. quintana* and *B. henselae* DNA amplified from blood and blood culture, but was *Bartonella* spp. non-seroreactive, potentially due to seroreversion associated with the long course of antibiotics. Despite differences in age, there was a considerable overlap in neurological symptoms reported by the father, oldest daughter, and son. Neurobartonelloses are being associated with an expanding spectrum of neurological presentations [43,44]. The results of this investigation provide additional support for prospective studies that investigate the potential role and interactions of stealth vector borne pathogens as a cause of neuropsychiatric presentations.

There were several limitations to this outbreak investigation. Due to extensive travel, vector exposure histories, as well as the frequent fostering of dogs from diverse locations, establishing the timing and source of infection for each individual family member or pet was not possible. Serological and molecular research test results, particularly for the son and youngest daughter, were potentially influenced by prior antibiotic therapy. Also, detection of stealth *Babesia* and *Bartonella* infections does not confirm that these infections were contributing to symptoms in family members, or the pathologies documented in the pet dog. Our research results were reported to attending physicians who had sole responsibility for determining if treatment interventions were indicated. As laboratory research testing was performed over a period of years, the medical implications remain unclear, despite documenting a potential role for stealth pathogen infection in family members, pets, and flea vectors.

## 10. Conclusions

Results from this investigation reinforce the importance of obtaining a chronological and comprehensive medical history, including animal and arthropod exposures, and travel history for both people and their pets. A One Health outbreak investigation approach should ensure a more holistic understanding of disease transmission risks among animals and human patients, whereby investigational results could potentially contribute to better patient outcomes or improved prevention strategies. To achieve this objective, both strategically designed infrastructure and financial support of One Health investigational approaches are needed.

## Figures and Tables

**Table 1:** Symptoms reported on a structured questionnaire by the five family members. “Yes” indicates the associated box was checked whereas—indicates the box was not checked by the study participant. Age (in years) of each family member at the time of research blood sample submission in 2022 is listed.

Self- or Parent-Reported Symptoms	Father 43 Years Old	Mother 42 Years Old	Oldest Daughter 14 Years Old	Youngest Daughter 13 Years Old	Son 10 Years Old
Blurred vision	Yes	Yes, ocular migraines	-	Yes	Yes
Headache	Yes	-	Yes	Yes	-
Mental confusion	Yes	-	Yes	-	Yes
Memory loss	Yes	-	Yes	-	Yes
Anxiety/Panic attacks	Yes	-	-	Yes, intense fear of wind	Yes
Fatigue	Yes	-	Yes	-	-
Insomnia	Yes	-	Yes	-	-
Irritability/Rage/Aggression	Yes	-	-	-	Yes
Depression	Yes	-	-	-	Yes
Paresthesia	Yes, legs	-	-	-	Yes, arms
Joint pain	Yes, hips	-	-	Yes, elbows and ankles	-
Weight gain	-	-	Yes	-	Yes
Shortness of breath	Yes	-	-	-	-
Neck pain	Yes	-	-	-	-
Tremors	-	-	Yes	-	-
Vertigo	-	-	-	Yes	-
Hallucinations	-	-	-	-	Yes
Eye pain	-	-	-	-	Yes
Muscle pain	-	-	-	-	Yes, legs

**Table 2:** *Bartonella* and *Babesia* qPCR (confirmed by DNA sequencing) and ddPCR results for three pets owned by the family.

Family Pet	Sample	*Bartonella*		*Babesia*	
		qPCR	ddPCR	qPCR	ddPCR
Dog 1	Spleen	*B. henselae*	NEG	*B. divergens*-like MO-1	POS
	Skin	*B. quintana*	NEG	NEG	EQUIV
	Blood culture (C14)	*B. quintana*	NEG	NEG	NEG
Dog 2	Blood culture (C7)	*B. quintana*	EQUIV	NEG	EQUIV
Rabbit	Blood	*Bartonella* spp.	EQUIV	NEG	EQUIV

Digital PCR results reported as negative (NEG, no dots), equivocal (EQUIV, 1–2 dots), positive (POS, 3 or more dots).

**Table 3:** *Bartonella* species indirect fluorescent antibody titers and *Bartonella* qPCR, dPCR species probes, and DNA sequencing results for each family member.

Family Member	*Bartonella* IFA Reciprocal Titers (Serum)					*Bartonella* PCR/DNA Sequencing (Blood)			
						Direct Extraction		Enrichment Culture (Days)	
	*Bh*SA2	*Bvb* I	*Bvb* II	*Bk*	*Bq*	qPCR	dPCR	qPCR	dPCR
Father	1:256	1:128	1:128	1:256	1:128	EQUIV	*Bq*	NEG	*Bq* (C7)
Mother	1: <16	1: <16	1: <16	1: <16	1: <16	NEG	*Bq*	*Bq*-like (C7)	*Bq* (C7)
Oldest Daughter	1:128	1:32	1:64	1: <16	1: <16	NEG	*Bh* *Bq*	EQUIV (C21)	NEG
Youngest Daughter	1:128	1:64	1:128	1: <16	1:128	EQUIV	NEG	*Bh* (C7)	*Bq* (C7)
Son	1:16	1: <16	1: <16	1: <16	1:32	EQUIV	*Bq*	*Bh* (C14)	*Bh* (C14) *Bq* (C7,14,21)

*BhSA2*: *Bartonella henselae* strain San Antonio 2; *Bvb* I or II: *Bartonella vinsonii* subsp. *berkhoffii* genotype I or II; *Bk*: *Bartonella koehlerae*; *Bq*: *Bartonella quintana*.

**Table 4:** *Babesia* molecular testing results for each family member.

Family Member	Sample	18S rRNA		ITS qPCR		
		qPCR	dPCR	*Babesia* Species	% Homology (bp)	Compared GenBank Sequence Accession
Father	Blood	EQUIV	NEG	*B. divergens* like MO-1	99.2 (614/619)	PQ18454
	Blood Culture (C7)	NEG	POS	*B. microti* PQ459010 *	99.6 (475/477)	AB190459
Mother	Blood	NEG	NEG	*B. microti*	99.5 (386/388)	AB190459
	Blood Culture (C7)	EQUIV	POS	*B. divergens* like MO-1 *B. microti*	95.1 (372/391) 99.4 (538/540)	PQ184854 AB190459
Oldest Daughter	Blood Culture (C7)	NEG	EQUIV	*B. divergens* like MO-1	99.4 (1020/1026)	PQ184854
	Serum	NEG	POS	*B. divergens* like MO-1	99.4 (924/930)	PQ184854
Youngest Daughter	Blood Culture (C14)	NEG	EQUIV	*B. divergens* like MO-1	99.4 (1039/1045)	PQ184854
Son	Blood Culture (C14)	NEG	NEG	*B. divergens* like MO-1 PQ459274 *	99.2 (615/620)	PQ18454
	Serum	NEG	NEG	*B. divergens* like MO-1	99.2 (617/622)	PQ18454

* Submitted GenBank sequence accession from this study.

## Data Availability

To assure patient confidentiality, please contact E.B.B. regarding data and materials. Data that references human results is unavailable due to privacy and ethical restrictions (IRB#s 4925-03 and 164-08-05). All other data are available upon request.
